# Supplementary material for: Inhalation of the prodrug PI3K inhibitor CL27c improves lung function in asthma and fibrosis
Source: Nat Commun. 2018 Dec 12;9:5232. doi: 10.1038/s41467-018-07698-6 (PMC6290777; doi:10.1038/s41467-018-07698-6)
Supplement: Supplementary file 1 — Supplementary Informations [file 41467_2018_7698_MOESM1_ESM.pdf]

Inhalation of the prodrug PI3K inhibitor CL27c improves lung function in asthma and fibrosis

Carlo C. Campa, Rangel L. Silva, Jean P. Margaria, Tracey Pirali, Matheus S. Mattos, Lucas R. Kraemer, Diego C. Reis, Giorgio Grosa, Francesca Copperi, Eduardo M. Dalmarco, Roberto C.P. Lima-Júnior, Silvio Aprile, Valentina Sala, Federica Dal Bello, Douglas Prado, Jose Carlos Alves-Filho, Claudio Medana, Geovanni D. Cassali, Gian Cesare Tron, Mauro M. Teixeira, Elisa Ciraolo, Remo C. Russo, Emilio Hirsch.

Supplementary Information

Supplementary Figures……………………………………………………………2
Supplementary Tables……………………………………………………………24
Supplementary Methods…………………………………………………………....29

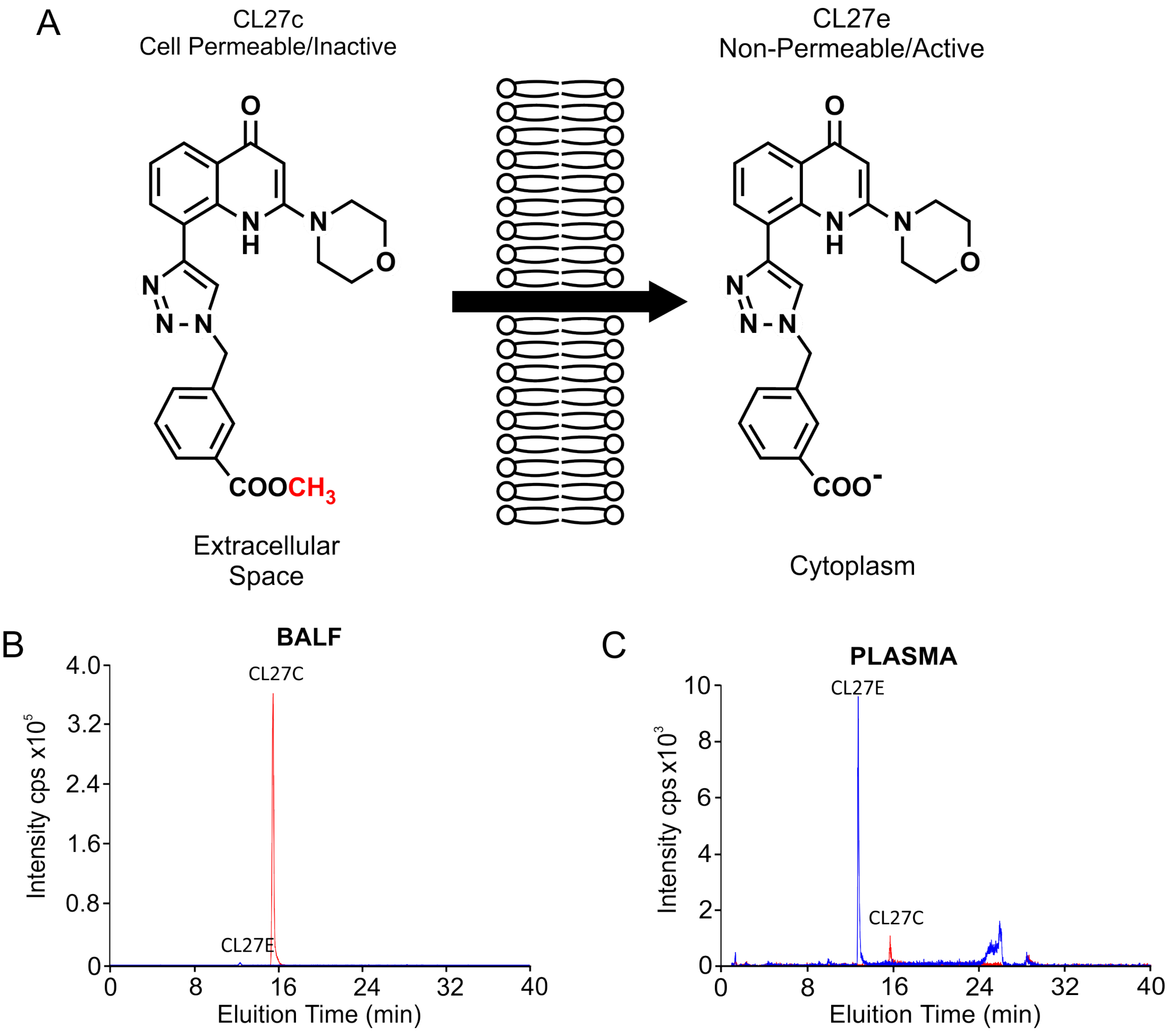

Supplementary Figure 1: CL27c is converted into the active compound CL27e.

(A) Chemical structures of prodrug CL27c and its intracellular pharmacologically active metabolite, CL27e. Note the methyl ester (red) is hydrolyzed once the prodrug passes through the plasma membrane (arrow). (B-C) quantification of CL27c in mouse plasma (B) and BALF (C) through HPLC coupled with tandem mass spectrometry. B and C are the chromatograms where the showed picks correspond to CL27c and CL27e detection, respectively.

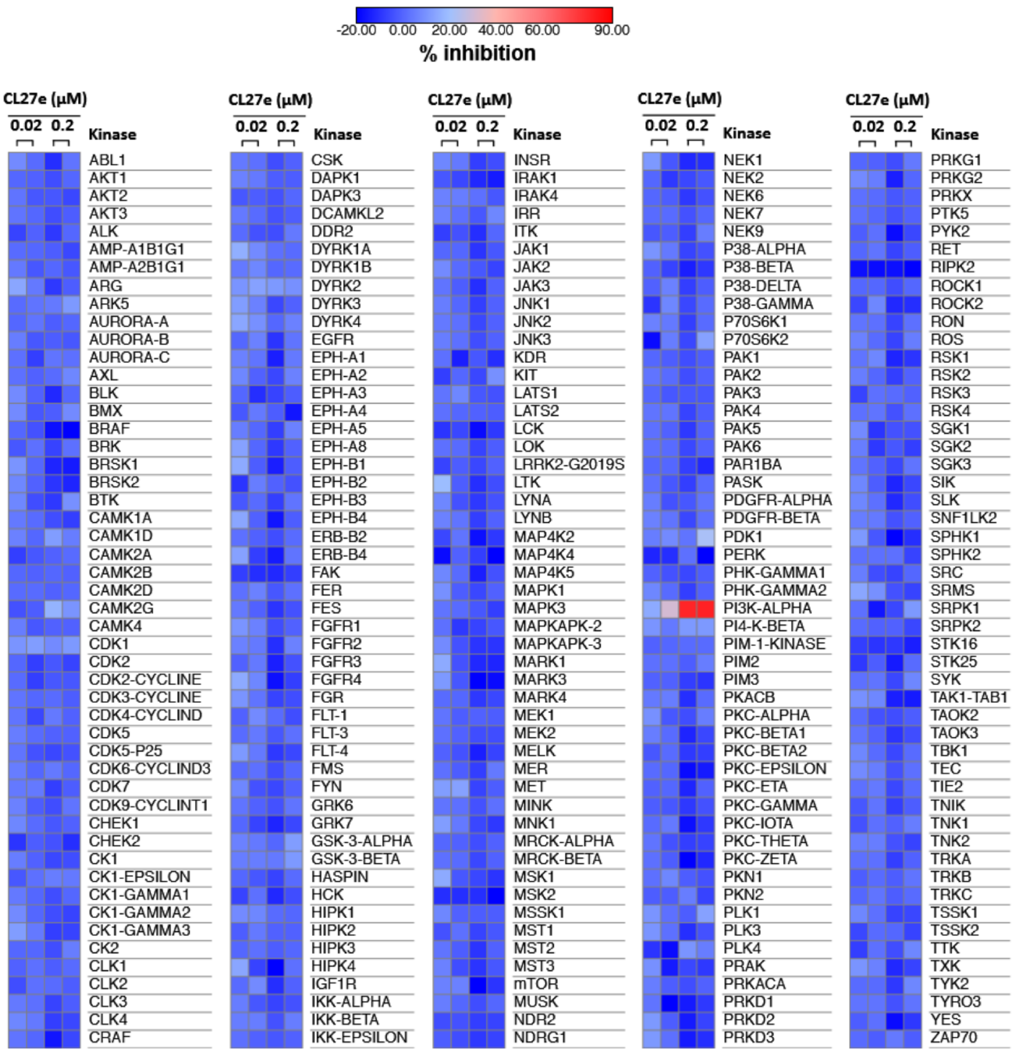

Supplementary Figure 2: Kinase selectivity of CL27e

Kinase selectivity of the CL27c active metabolite, CL27e, was evaluated at 0.02 µM and 0.2 µM using the Nanosyn 250-kinase panel. Red color indicates selectivity for PI3Kα, on which CL27e showed >80% inhibition at 0.2 µM. Results are expressed as mean percentage inhibition for each concentration .

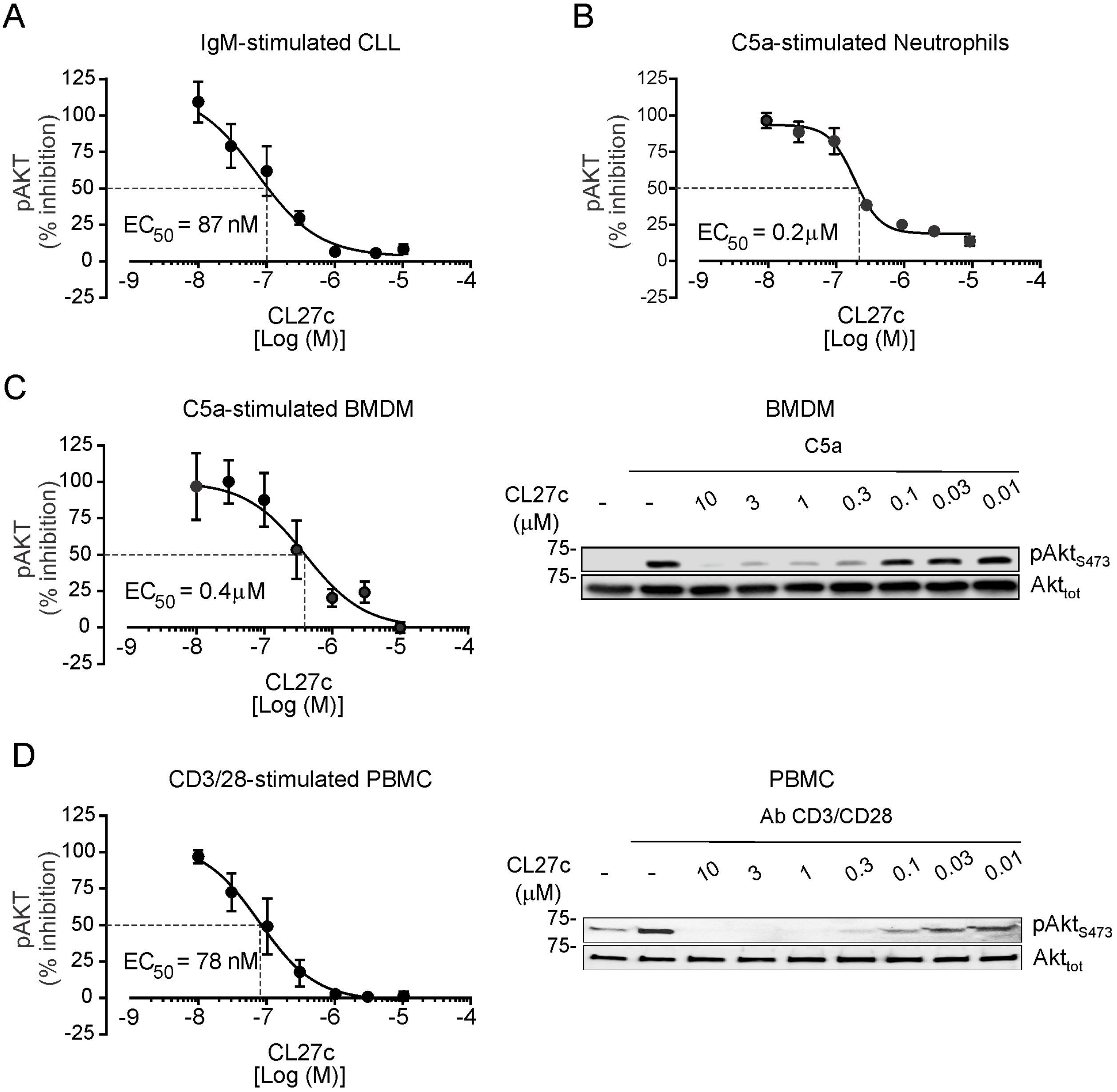

Supplementary Figure 3: Inhibition of Akt phosphorylation at Ser473 ($pAkt_{473}$)

(A) Dose response analysis of CL27c (10 nM – 10 μM) in human CLL cells stimulated with IgM (20 μg/ml; n=3; representative blot is in Fig. 1A). (B) Dose response analysis of CL27c in mouse primary neutrophils stimulated with C5a (25 nM; representative blot is in Fig. 1A). (C) Dose response analysis of CL27c in murine primary bone marrow-derived mononuclear cells (BMDM) stimulated with C5a (25 nM). (D) Dose response analysis of CL27c in human PBMC stimulated with CD3/CD28 antibodies (1μg/ml; n=3) and representative western blot (right

panel). Results represent mean ± s.e.m. of replicates n=3 for each group. Each dose-response curve was obtained by plotting the % of $pAkt_{473}$ over control (cells treated with vehicle) versus logarithmic concentration of the inhibitor. The respective EC_{50} was calculated by using nonlinear regression fit.

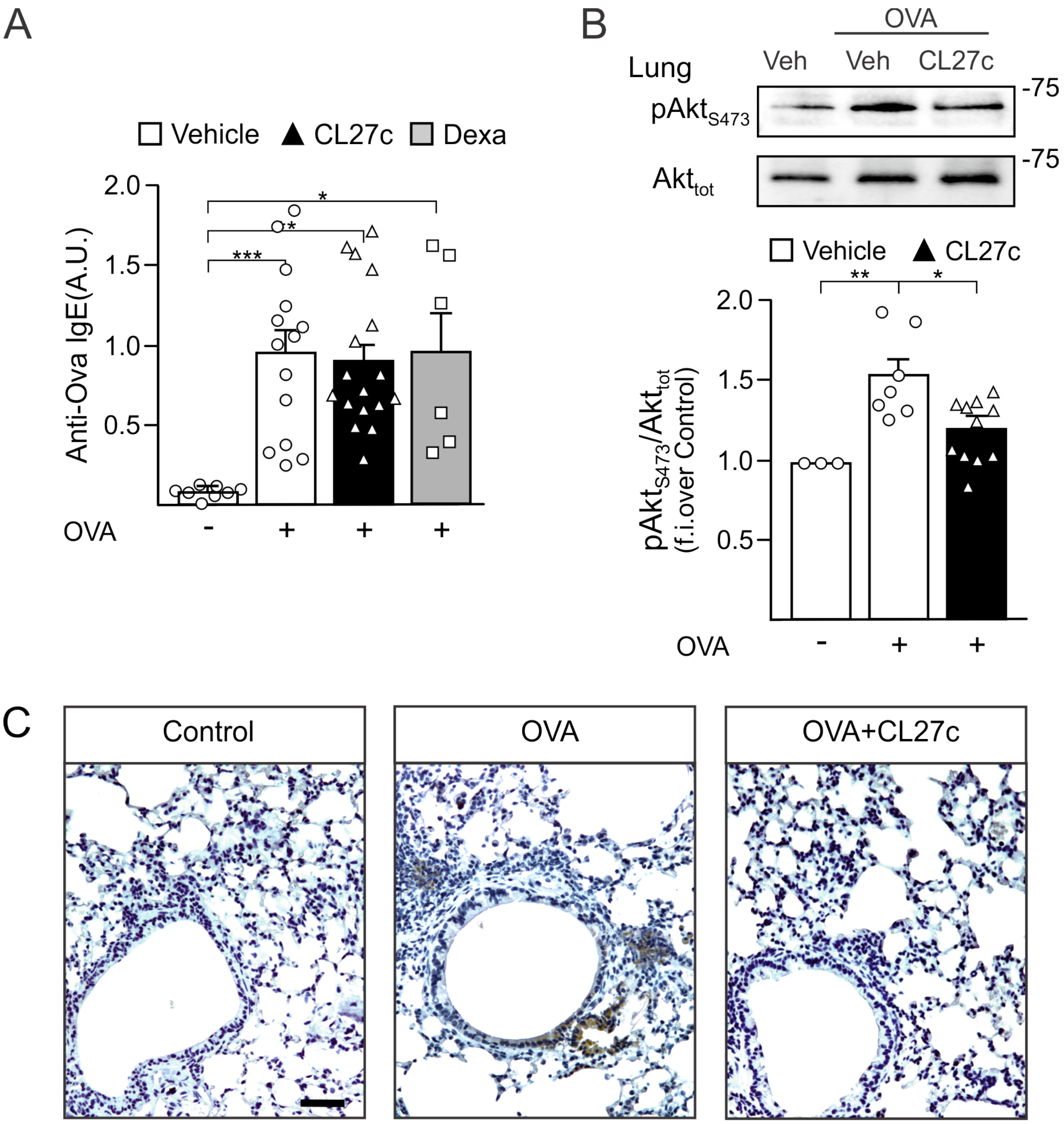

Supplementary Figure 4: Validation of the disease model and target engagement.

(A) Analysis of anti-OVA IgE production in immunized mice, challenged with OVA following the scheme showed in Fig. 2 (from left to right n= 8, 14, 16, 6 independent experiments respectively). (B) Western blot analysis of PI3K pathway inhibition. Upper panel: representative western blot of PI3K-dependent Akt phosphorylation at Ser473 ($pAkt_{473}$). Lower panel: statistical analysis of $pAkt_{473}$ /Akt_{tot} (total Akt) over the unchallenged control (from left to right n= 3, 7, 11 independent experiments respectively). Results represent mean±s.e.m. , *P<0.05, **P<0.01, ***P<0.001 determined using one-way ANOVA followed by Bonferroni post-hoc

test. (C) Representative immunohistochemistry for $pAkt_{473}$ in lung sections from control mice or animals treated with OVA alone or in combination with CL27c. Bar: 100μM.

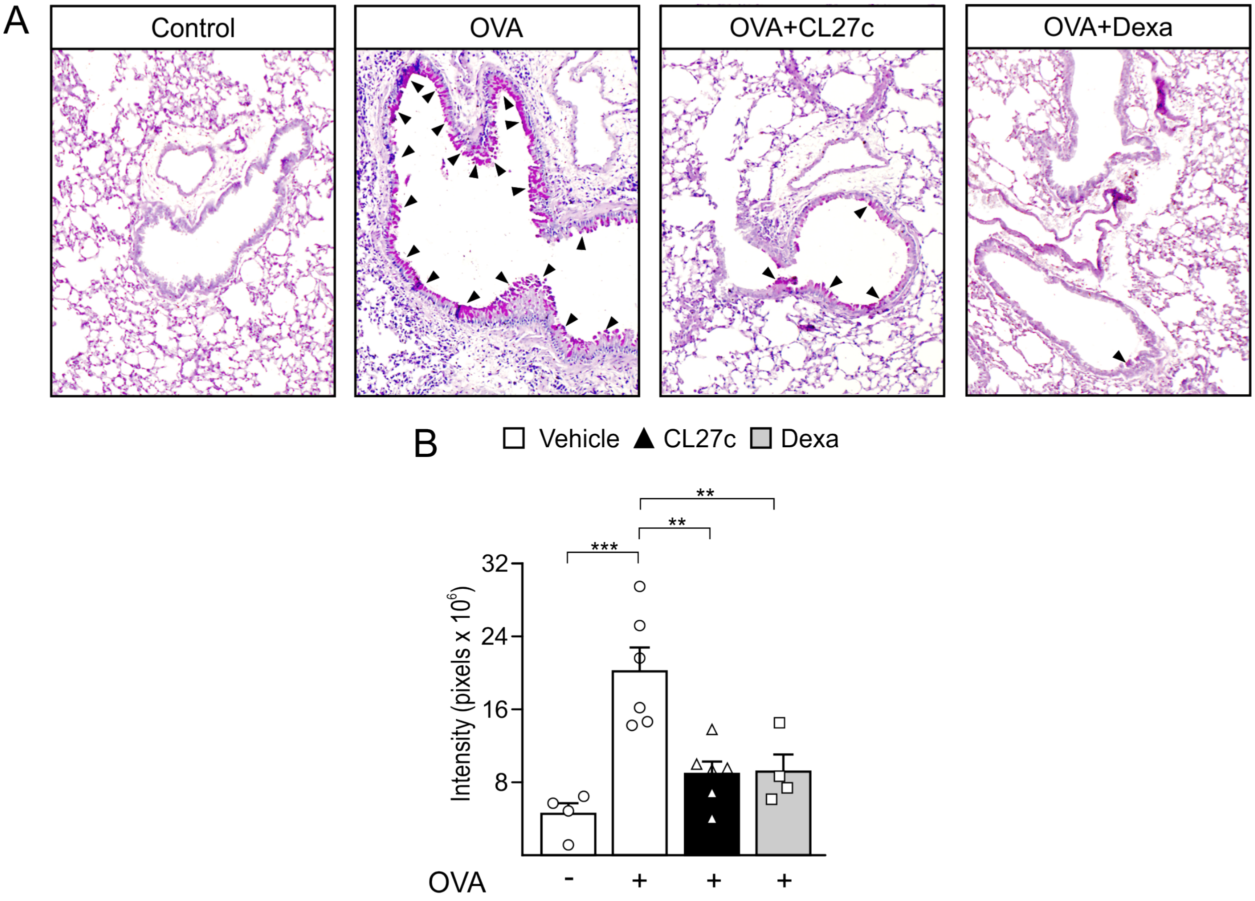

Supplementary Figure 5: CL27c prevents Ovalbumin (OVA)-induced chronic allergic response induces metaplasia of goblet cells and mucus production

(A) Analysis of lung sections stained with periodic acid-Schiff (PAS). Arrowheads indicate PAS-positive cells in the bronchiolar area. (B) Quantification of PAS staining intensity in mice challenged with OVA and treated with vehicle, CL27c or Dexamethasone (from left to right n= 4, 6, 6, 4 independent experiments respectively). Results represent mean±s.e.m. , $^{**}P<0.01$, $^{***}P<0.001$ determined using one-way ANOVA followed by Bonferroni post-hoc test.

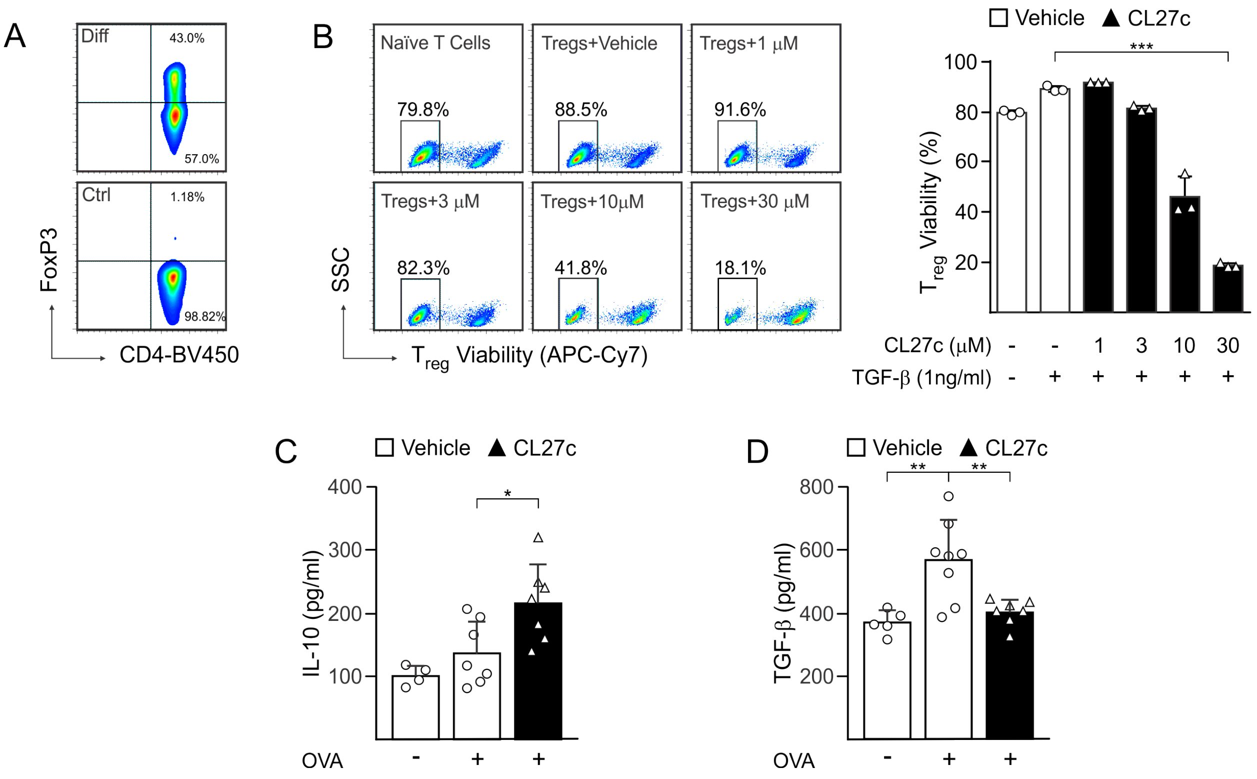

Supplementary Figure 6: Effects of CL27c on Tregs

(A) Representative FACS analysis of Treg differentiation in the presence of TGF-β1. (B) FACS analysis for the viability of *in vitro* differentiated Treg after incubation with the given concentrations of CL27c. Left panel: representative FACS image for the viability versus side scatter (SSC). Right panel: statistical analysis of Treg viability after treatment with CL27c (n= 3, independent experiments). (C) Measurement of IL-10 concentration in lung tissue lysates of control and OVA challenged mice as described in Figure 2 in the presence or absence of inhaled CL27c (from left to right n= 4, 7, 7 independent experiments respectively). (D) Measurement of TGF-β concentration in lung tissue lysates of control and OVA challenged mice as described in Fig. 2 in the presence or absence of inhaled CL27c (from left to right n= 5, 8, 7 independent experiments respectively). Results represent mean±s.e.m. , * $P<0.05$, ** $P<0.01$, *** $P<0.001$ determined using one-way ANOVA followed by Bonferroni post-hoc test.

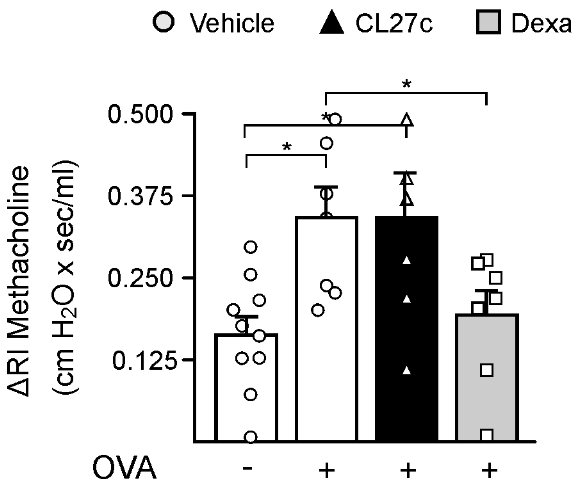

Supplementary Figure 7: CL27c failed to prevent methacholine-induced airway hyperresponsiveness during chronic asthma

Analysis of airway hyperresponsiveness after treatment of Methacholine in animals with OVA-induced chronic asthma after the treatment with vehicle, CL27c and dexamethasone (Dexa) (from left to right n= 10, 7, 6, 7 independent experiments respectively). Results represent mean±s.e.m. , *P<0.05 determined using one-way ANOVA followed by Bonferroni post-hoc test.

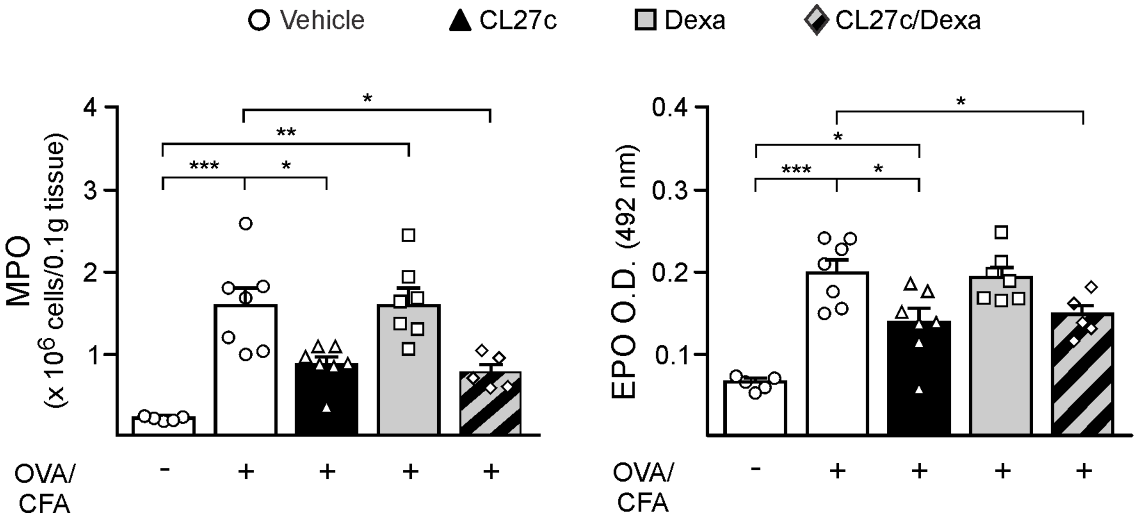

Supplementary Figure 8: Analysis of Myeloperoxidase (MPO)

(A) and eosinophil peroxidase (EPO) (B) in mice treated with CL27c alone or combined with dexamethasone (Dexa) (from left to right groups, MPO n= 5, 7, 7, 7, 5, EPO n= 5, 7, 7, 7, 5 independent experiments respectively). Results represent mean±s.e.m. , *P<0.05, **P<0.01, ***P<0.001 determined using one-way ANOVA followed by Bonferroni post-hoc test.

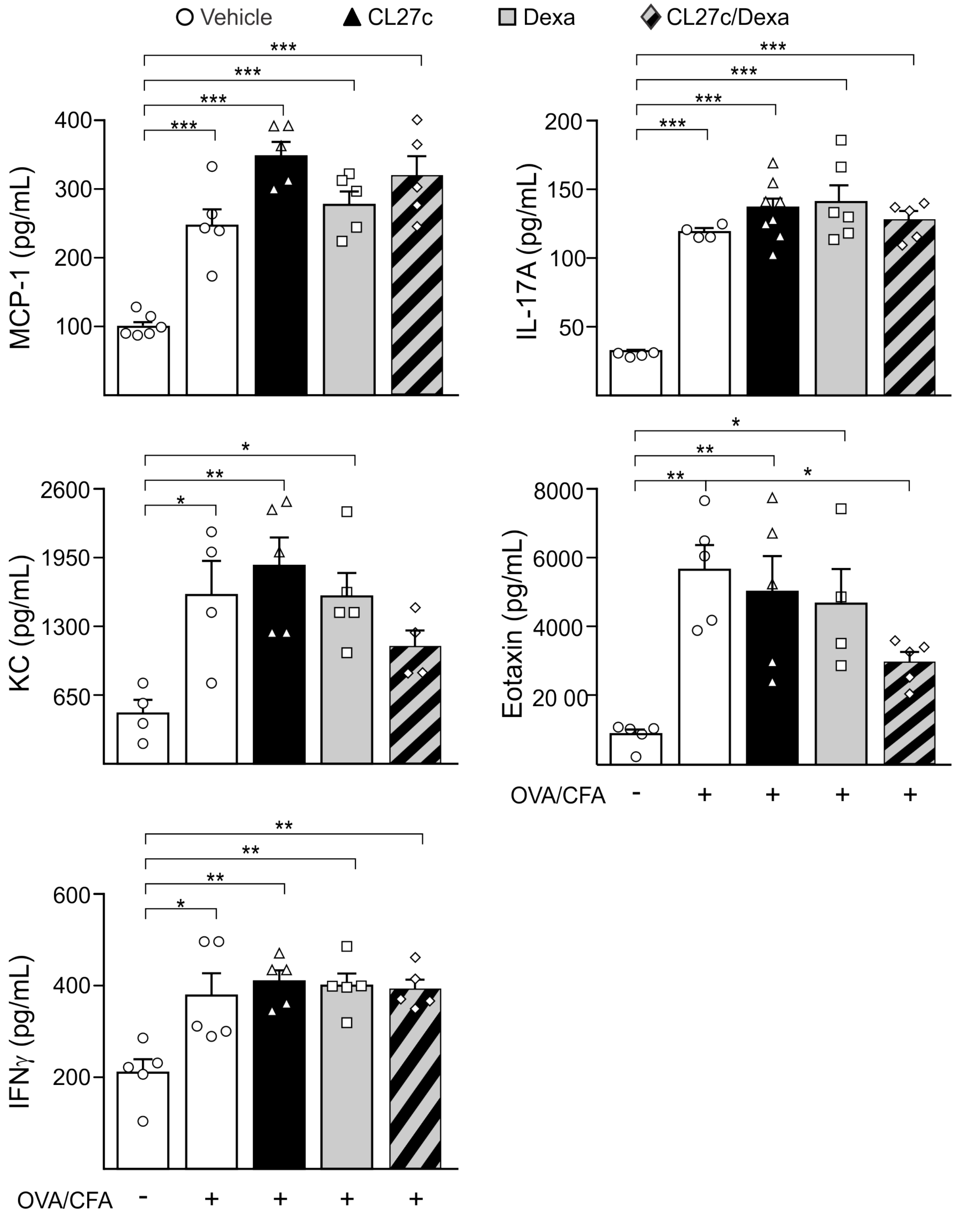

Supplementary Figure 9: Effects of CL27c in a model of corticosteroid-resistant asthma

(A) Quantification of MCP-1, IL-17A, KC, Eotaxin and IFN-γ levels in lung tissues (from left to right MCP-1 n= 6, 5, 5, 5, 5, IL17A n= 4, 4, 8, 6, 5, KC n= 4, 4, 5, 5, 4, Eotaxin n= 5, 5, 5, 4, 5,

IFNγ 5, 5, 5, 5, 5 independent experiments respectively). Results represent mean±s.e.m. , *$P<0.05$, **$P<0.01$, ***$P<0.001$ determined using one-way ANOVA followed by Bonferroni post-hoc test.

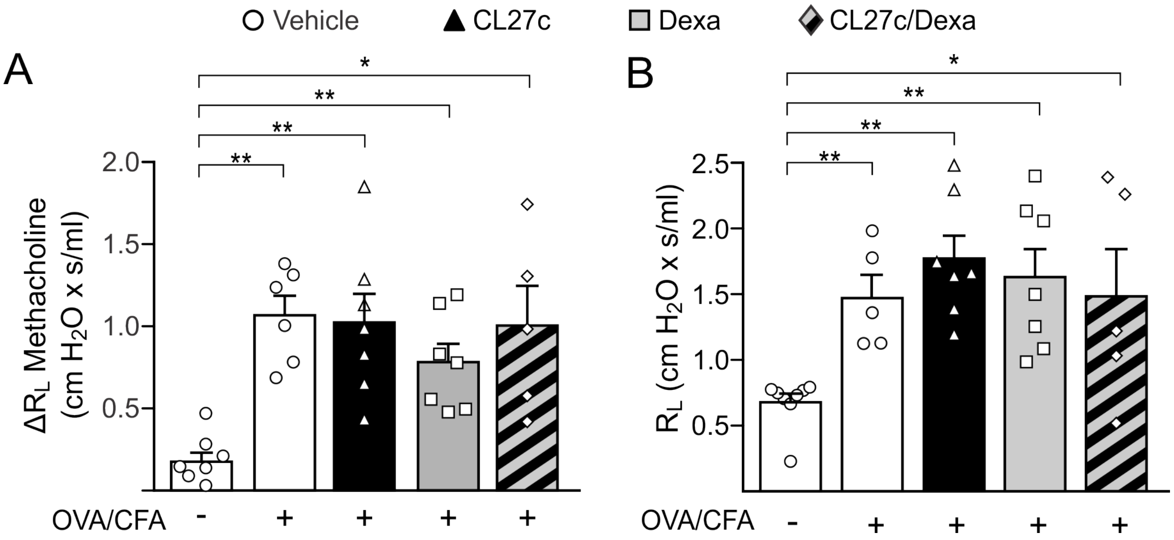

Supplementary Figure 10: **Lung physiology in a model of corticosteroid-resistant asthma**
(A) Analysis of bronchial hyperresponsiveness to methacholine (from left to right n= 7, 6, 6, 7, 5 independent experiments respectively). (B) Measurement of inspiratory resistance (from left to right n= 8, 5, 7, 7, 5 independent experiments respectively). Results represent mean±s.e.m. , $*P<0.05$, $**P<0.01$ determined using one-way ANOVA followed by Bonferroni post-hoc test.

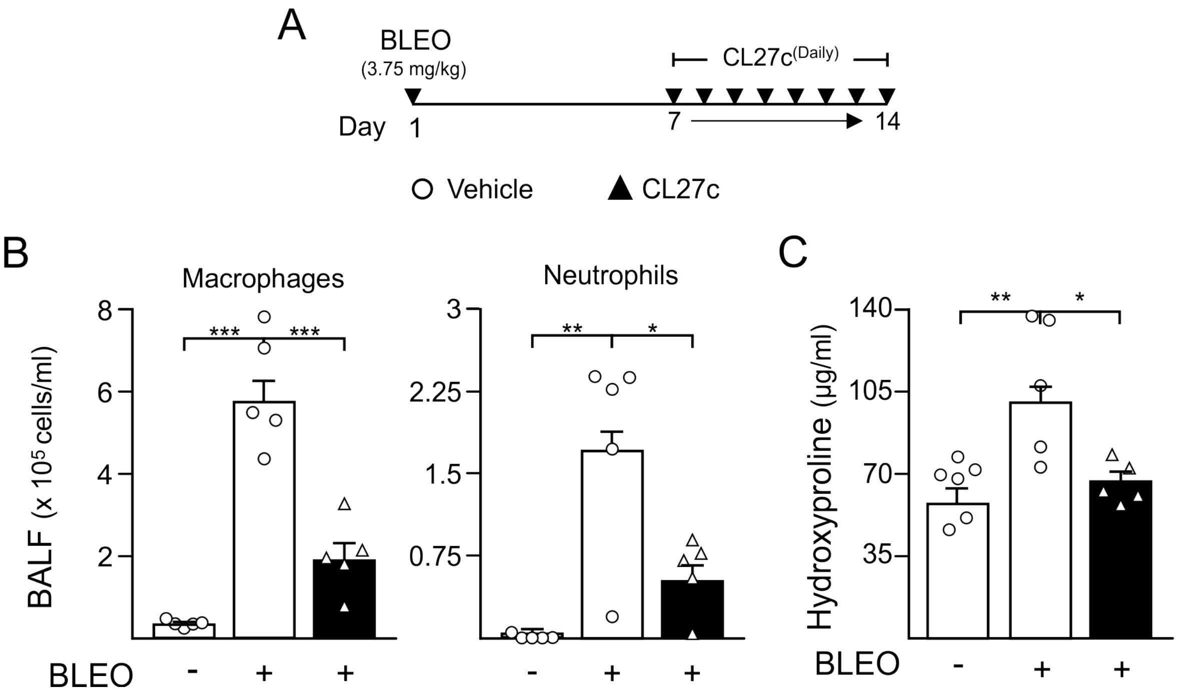

Supplementary Figure 11: **Inhaled CL27c decreases inflammation and fibrosis in a model of severe bleomycin-induced lung fibrosis**

(A) Severe protocol of bleomycin-induced (3.75 mg/kg) lung fibrosis. (B) Analysis of macrophages and neutrophils infiltration in BALF in the presence (2 mg/ml suspension) or absence of CL27c (from left to right, macrophages n= 5, 5, 5 and neutrophils n= 5, 5, 5 independent experiments respectively). (C) Quantification of hydroxyproline deposition in the presence or absence of CL27c (from left to right n= 6, 5, independent experiments respectively). Results represent mean±s.e.m. , *P<0.05, **P<0.01, ***P<0.001 determined using one-way ANOVA followed by Bonferroni post-hoc test.

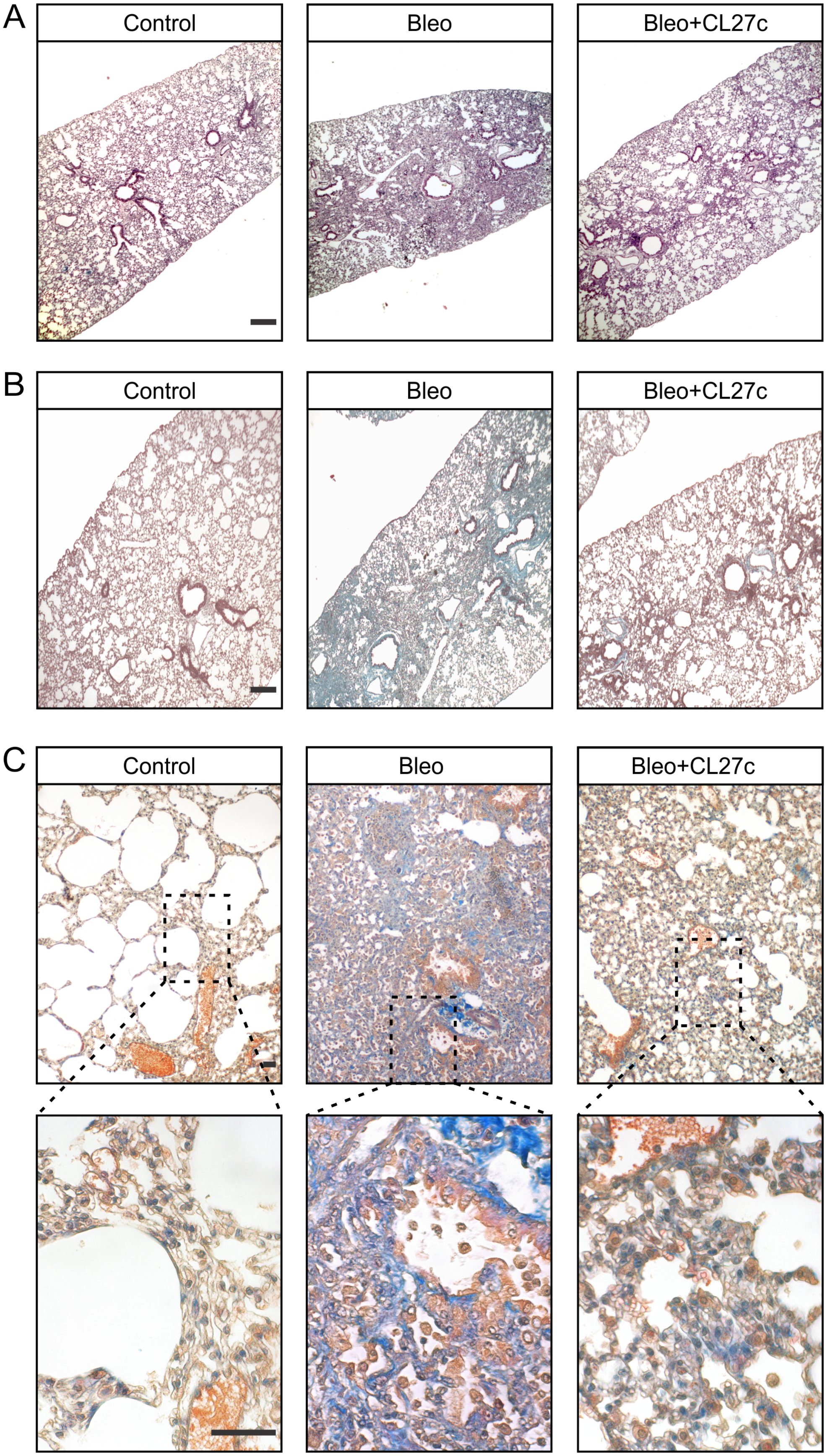
A
Control
Bleo
Bleo+CL27c
B
Control
Bleo
Bleo+CL27c
C
Control
Bleo
Bleo+CL27c

Supplementary Figure 12: **Aerosolized CL27c treatment decreases tissue damage and collagen deposition in a model of severe fibrosis induced by bleomycin (2 mg/kg)**

(A) Representative sections stained with hematoxylin and eosin. (B, C) Representative sections stained with Gomoris's trichrome (B) and Masson's trichrome (C), evidencing fibrotic areas in green and blue, respectively. Bars represent 500 μM (A, B) and 100 μM (C).

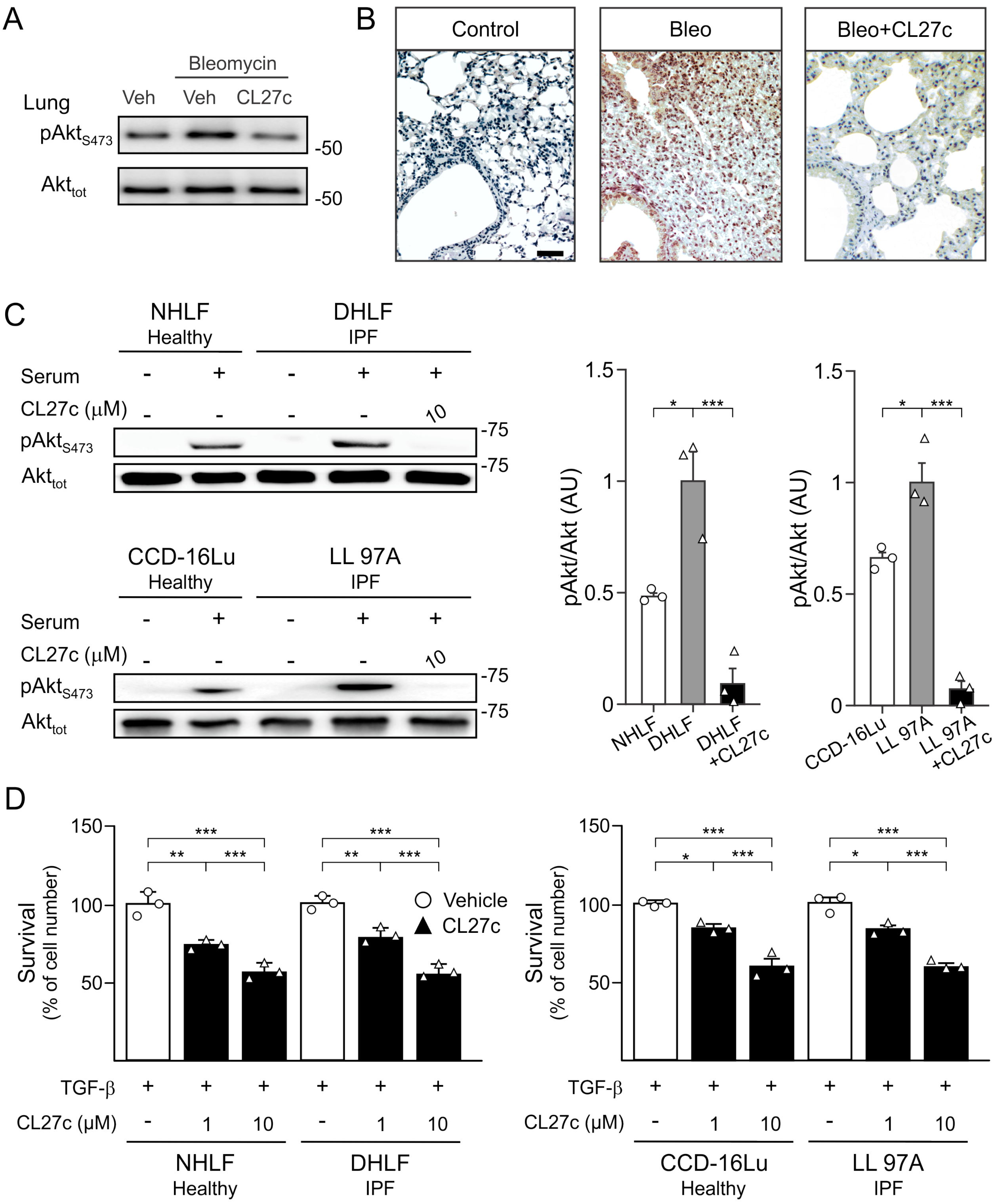

Supplementary Figure 13: Effects of CL27c on human healthy and IPF lung fibroblasts Bleomycin (3.75 mg/kg) was used to induce lung fibrosis in mice. (A) Representative western

blot analysis of $pAkt_{473}$ in lungs from control and bleomycin-treated mice alone, or in the presence of CL27c. (B) Immunohistochemical analysis of $pAkt_{473}$ in lung sections as in A. (C) Western blot analysis of AKT phosphorylation ($pAkt_{473}$) after serum stimulation of healthy or IPF human lung fibroblast treated with vehicle or CL27c (10 µM). One representative blot is shown of three independent experiments. Bar charts on the right show quantification of phospho-AKT ($pAkt_{473}$) compared to total AKT (Akt_{tot}) control in serum stimulated healthy, IPF and CL27c-treated IPF fibroblasts. (D) Survival of primary human lung fibroblasts (HLF) and immortalized HLF cultured with TFG-β (10 ng/ml) and kept for 48 hours in the absence or presence of CL27c (1 µM or 10 µM). Data are expressed in % of cells in comparison to day 1. Bar: 100µM. Results represent mean±s.e.m. of n= 3 for each group. *P<0.05, **P<0.01, ***P<0.001 determined using two-way ANOVA followed by Bonferroni post-hoc test.

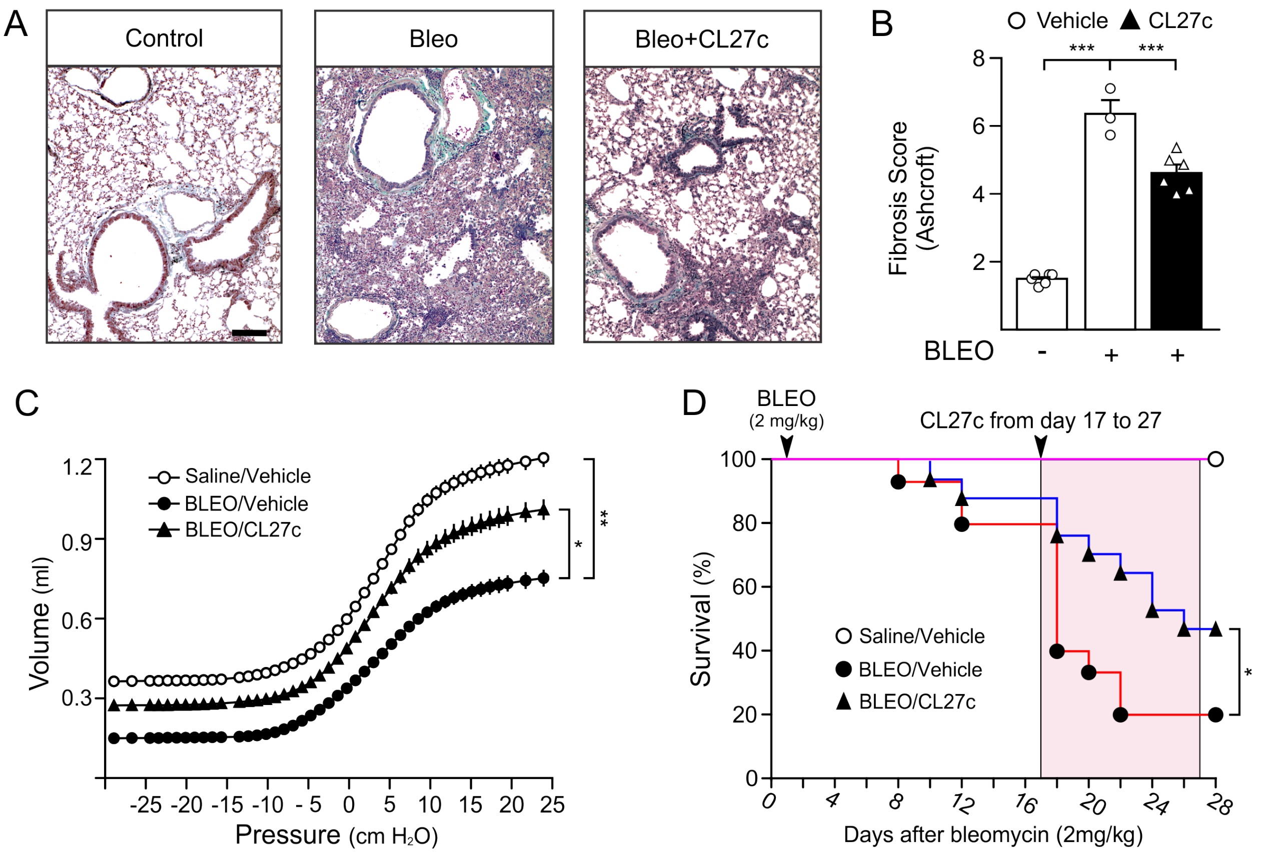

Supplementary Figure 14: Effects of inhaled CL27c treatment starting at day 17 after Bleomycin (2 mg/kg) administration

(A) Quantification of fibrosis in control mice and animals treated with bleomycin alone or in the presence of CL27c. Bar: 300µM. (B) Quantification of fibrosis on lung sections using the semi-quantitative Ashcroft scale (from left to right n= 6, 3, 6 independent experiments respectively). (C) Analysis of the Pressure Volume curve in control mice (n=15) and in mice that developed Bleomycin-induced lung dysfunction after treatment with vehicle (n=5) or CL27c (n=7; 2 mg/ml). (D) Survival curves of mice treated with either saline (white circles; n=6) or Bleomycin in the presence (black triangle; n=17) or absence (black circles; n=15) of CL27c. Results represent mean ± s.e.m. , *P<0.05, **P<0.01, ***P<0.001 determined using one-way ANOVA followed by Bonferroni post-hoc test. Animal survival was analyzed by the Mantel-Cox log rank test.

Figure 1a

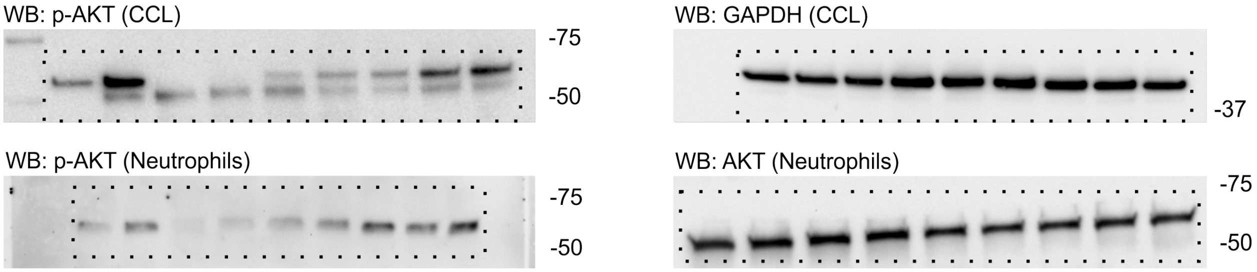

Figure 1e

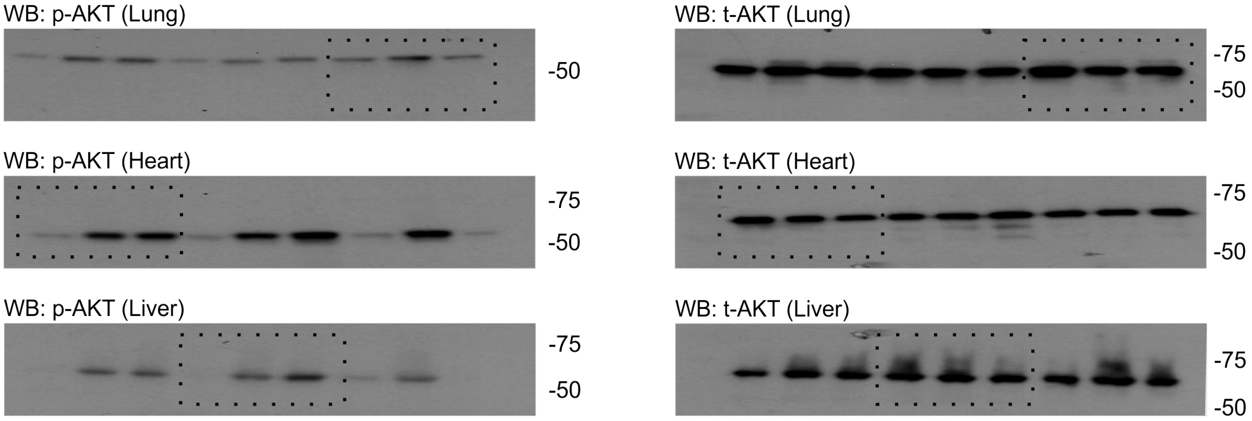

Figure S3c

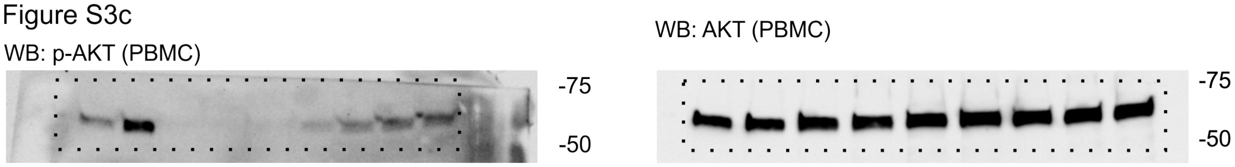

Figure S3d

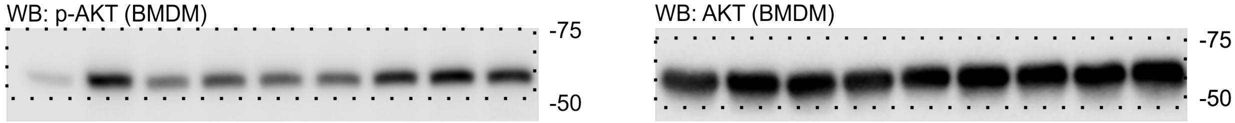

Figure S4b

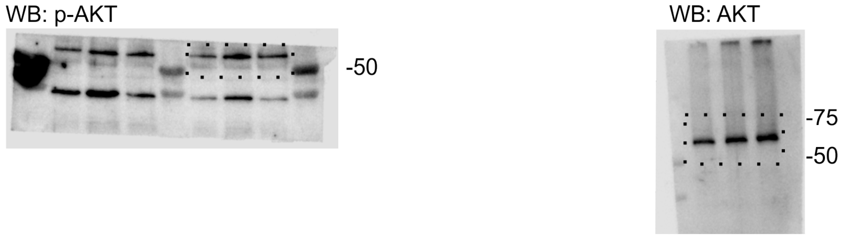

Figure S13a

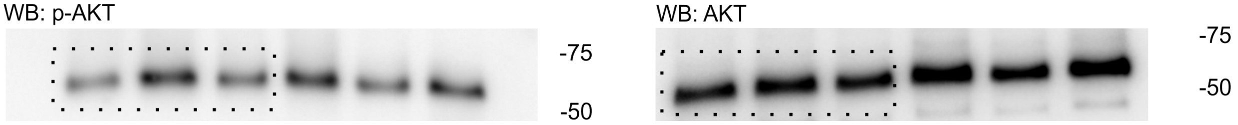

Figure S13c

WB: p-AKT

WB: AKT

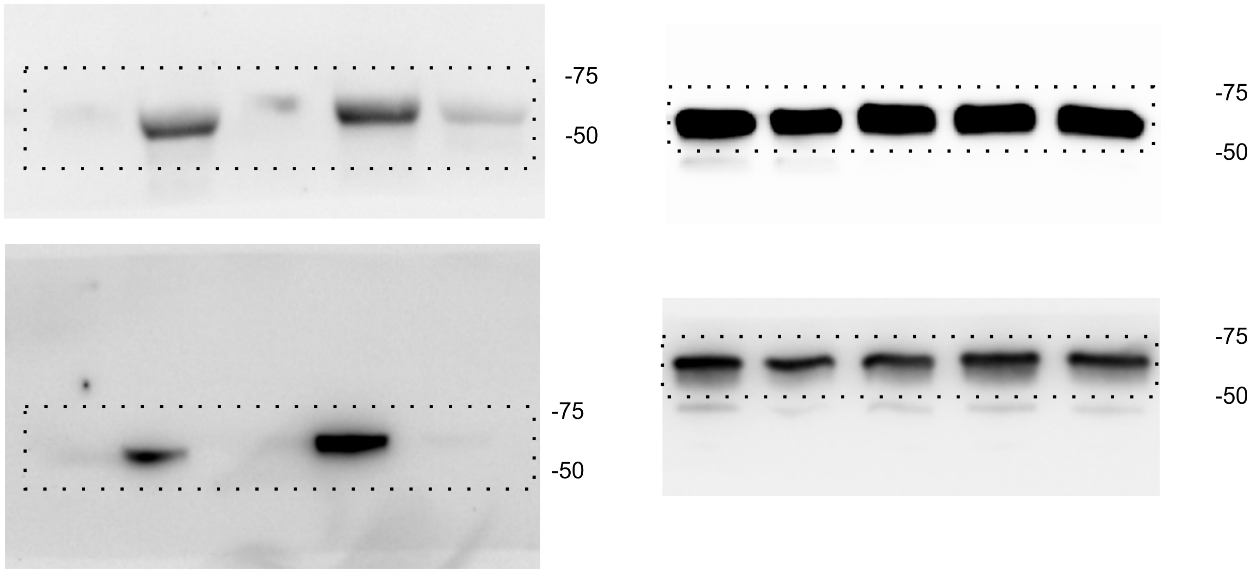

Supplementary Figure 15: **Lists of original gel images of western blot analysis**

Boxes highlight lanes used in figures. To minimize antibody loss and to allow concomitant detection of more than one protein on the same gel, nitrocellulose filters have been cut with a knife at the specified molecular weights. This technique avoided stripping and comparison of protein content loaded on different gels. Only stripes were further processed and this explains why figures might look incomplete. Please note the darker horizontal shadows corresponding to top and bottom of filters.

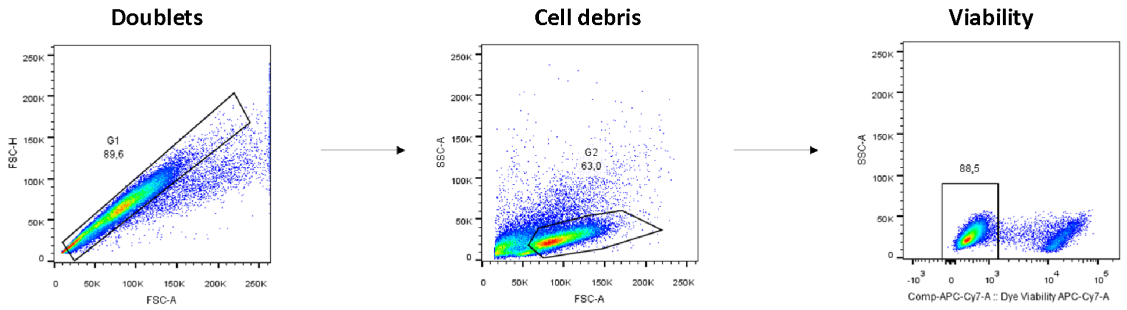

Supplementary Figure 16: **Representative gating strategy used for flow cytometry analysis of cell viability**

Doublets were excluded by FSC-H and FSC-A gating (G1) and cell debris were excluded by SSC-A and FSC-A gating (G2) for all flow cytometry analysis. Proportion of viable cells stained with fixable viability dye APC-Cy7.

Supplementary Table 1. In vitro plasma half-life (t1/2), (mouse plasma).

		CL27c		CL27e	
Time points	(min)	0	30	0	30
Concentration	(ng/ml)	113.2 ± 12.8	6.2 ± 2.8	45.3 ± 14.1	43.6 ± 7.4
Recovery	%	100	5	100	96

Values are the mean of at least triplicate determination in a single experiment.

Supplementary Table 2. Microsomial stability.

	CL27c			CL27e		
Species	Mouse	Rat	Human	Mouse	Rat	Human
t1/2 (min)	12.4 ± 2.0	5.9 ± 0.1	10.2 ± 2.2	n.d.	n.d.	n.d.
Clint (ml/min/mg prot)	0.1 ± 0.02	0.2 ± 0.005	0.1 ± 0.03	n.d.	n.d.	n.d.

Legend: CLint, Intrinsic clearance; n.d., not determined

Values are the mean of at least triplicate determination in a single experiment.*Intrinsic clearance. n.d. not determined

Supplementary Table 3. Pharmacokinetic parameters of a single dose of CL27c

		CL27c			
		I.V. (10 mg/kg)		P.O. (50 mg/kg)	
Analyte		CL27c	CL27e	CL27c	CL27e
C_{max}	(mg/l)	0.93	1.90	0.22	0.29
T_{max}	(h)	0.08	0.08	0.5	0.5
AUC_{last}	(mg/l*h)	0.87	0.98	0.15	0.26
AUC_{tot}	(mg/l*h)	0.87	1.05	0.39	0.28
t½	(h)	0.65	1.25	1.05	0.43
CL	(ml/min/kg)	191	191	n.d.	n.d.
V_z	l/kg	10.75	10.75	n.d.	n.d.
V_{ss}	l/kg	13.55	13.55	n.d.	n.d.
F	%	100	91	3.40	4.80

Legend:I.V,. Intravenous administration; P.O., Intravenous administration; C_{max}, Maximum (peak) plasma drug concentration; AUC_{last} Area under the plasma concentration-time curve from time zero to time of last measurable concentration; AUC_{tot} Area under the plasma-concentration time curve; t½, Elimination half-life: CL, Apparent total body clearance of CL27c from plasma; V_z, Apparent volume of distribution during terminal phase; V_{ss}, Apparent volume of distribution at steady state;F, Bioavalability.

Supplementary Table 4. Histopathological Scoring System for mouse lungs

**Score 1)**	
**Airways Inflammation**	**Score /4**
Absent - (lack of inflammatory cells around airways)	0
Mild - (some airways have small number of cells)	1
Moderate - (some airways have significant number of cells)	2
Marked - (majority of airways have some inflammation)	3
Severe - (majority of airways are significant inflammed)	4
**Score 2)**	
**Vascular Inflammation**	**Score /4**
Absent - (lack of inflammatory cells around vessels)	0
Mild - (some vessels have small number of cells)	1
Moderate - (some vessels have significant inflammation)	2
Marked - (majority of vessels have some inflammation)	3
Severe - (majority of vessels are significantly inflammed)	4
**Score 3)**	
**Parenchymal Inflammation (at 10Xmagnification)**	**Score /5**
< 1% affected	0
1-9% affected	1
10-29% affected	2
30-49% affected	3
50-69% affected	4
>70% affected	5
Total Score = Score 1 + Score 2 + Score 3	Score /13

Supplementary Table 5. Primer sequences for Real-Time PCR

Gene	Reverse (R)	Forward (L)
TGF-β	5’-CAGCAGCCGGTTACCAAG-3’	5’-TGGAGCAACATGTGGAACTC-3’
CTGF	5’AGCGGTGAGTCCTTCCAAAG-3’	5’-TTCATGATCTCGCCATCGGG-3’
Col1a1	5’-GCAGCTGACTTCAGGGATGT-3’	5’-CATGTTCAGCTTTGTGGACCT-3’
MMP-2	5’- GATCCGTGGTGAGATCTTCTTC-3’	5’-GGGAGCTCAGGCCAGAAT-3’

Supplementary Methods

Human lung fibroblast cell culture

Two separate human lung fibroblast cell lines isolated from IPF patients were used: LL97a derived from a 48 years-old male (ATCC, Manassas, USA) and DHLF-IPF (#CC-7231, Lonza, Switzerland). Control cell lines were CCD-16Lu, obtained from a 35 years old male that died of astrocytoma –a disease unrelated to IPF - (ATCC) and NHLF (#CC-2512; Lonza). LL97a and CCD-16Lu cells were cultured in Dulbecco's modified Eagles medium (DMEM, Sigma Aldrich, Dorset, UK). Media was supplemented with penicillin/streptomycin (100 U/ml, Thermo-Fischer, Germany) and 10% fetal bovine serum (FBS, Thermo-Fischer, Germany). Cells from Lonza were cultured in the FGM^{TM} (#CC-3132, Lonza). DHLF-IPF and the other three cells lines were cultured and utilized at passages 2-3 and 3-6, respectively, to limit passage dependent bias. For experiments, medium was replaced with serum free medium, for a 1 hour-long starvation.

Kinome profiling assay

The enzyme assay panel (Nanosyn Biology, Inc., Santa Clara, CA, USA) employs microfluidics capillary electrophoresis technology to measure the change in electrophoretic mobility of the substrate upon phosphorylation. CL27c active metabolite, CL27e, was plated at 100x of the screening concentrations (0.02 or 0.2 μM) to be tested in a single point screening against 250 kinases. The buffer components (Magnesium Buffer [100 mM HEPES pH7.5; 0.01% Triton X-100; 0.1% BSA; 5 mM MgCl2; 1 mM DTT; 10 μM Sodium Orthovanadate; 10 μM Beta-Glycerophosphate]; Manganese Buffer [100 mM HEPES pH7.5; 0.01% Triton X-100; 0.1% BSA; 5 or 10 mM MnCl2; 1 mM DTT; 10 μM Sodium Orthovanadate; 10 μM Beta-Glycerophosphate]; Manganese/Magnesium Buffer [100 mM HEPES pH7.5; 0.01% Triton X-100; 0.1% BSA; 5mM MgCl2; 1 mM MnCl2; 1 mM DTT; 20 μM Sodium Orthovanadate; 20 μM Beta-Glycerophosphate]) and assay conditions were adjusted based on the specific assay (Table S4). Briefly, enzyme buffer (5 μl/well) was added with ATP (at apparent Km for each kinase, Table S4) to the 384-well assay plate. Then, the compound (100 nl) was transferred to the assay plate (Labcyte Echo 550, San Jose, CA, USA), followed by the substrate buffer (5 μl/well). The reaction mixture was incubated at 25oC according to the assay specific incubation time. For

Caliper based assay, the reaction was terminated by addition of EDTA buffer. Substrate and product were separated and quantified electrophoretically using the microfluidic-based and the assay plate was read on Caliper LabChip 3000 Drug Discovery System from Caliper Life Sciences (Walthan, MA, USA). ADP Glo was another method of detection used for some kinase assays where data was obtained by quantifying the intensity of luminescence. For ADP Glo based assay, the reaction was terminated by applying the Promega ADP Glo Kinase Assay kit and the assay plate was read on a LJL Biosystems Analyst HT.

Measurement of AKT phosphorylation and cell survival of Human Lung Fibroblasts

Control and IPF HLF (described above) were seeded in a 96-well tissue culture plates at 1 x 10^5 cells/well (Falcon, Germany) and starved in serum-free medium for 2 hours. Protein extraction was performed 10 minutes after serum stimulation. AKT phosphorylation was measured by western blot, as described in the "Western blot analysis" in the Methods section. For cell survival studies, ells were treated with CL27c (1-10 μM) 30 min prior the addition of Recombinant Human TGF-β 10 ng/mL (Peprotech #AF-100-21C). Cell medium was changed daily with the reposition of the same treatments. CellTiter-Glo® Luminescent Cell assay (Promega, Madison, Wisconsin, EUA) were performed to measure cell proliferation after 48 h of cell culture following the manufacturer's instructions. Quantification was performed by using GloMax®-Multi Microplate Multimode Reader (Promega, Madison, Wisconsin, EUA).

Measurement of Mucus Production

Lungs were removed and fixed in Milloning buffer solution (pH 7.4) with 4% paraformaldehyde. For analysis of inflammation score around the bronchial region, the lung sections were stained with hematoxylin and eosin (H&E). Mucus production was analyzed from tissue sections stained with Harris hematoxylin stain and a combination of Periodic acid-Schiff (PAS) stain. Photomicrographs of airways obtained at 400 × magnification were analyzed using the software Image-Pro Plus (Image-Pro Plus, 4.1; Media Cybernetics, Houston, TX, USA). Nine to twelve bronchial areas per lung were outlined and quantified. Results were expressed as PAS positive area (pixels). Peribronchial fibrosis was analyzed from tissue sections stained with hematoxylin and eosin stain and a combination of Masson's trichrome stain. Photomicrographs of airways

obtained at 200 × magnification were analyzed using the software Image-J (NIH, USA). Results were expressed as extracellular matrix deposition area (pixels).

Microsomial stability

All tests for the microsomial stability of CL27c were conducted by the Istituto di Ricerche Biomediche (RBM) SpA – Merck Serono, Ivrea, Italy.

PBMCs measurement of Akt phosphorylation

PBMCs in RPMI medium were seeded in a 96-well tissue culture plates at 1 x 10^5 cells/well (Falcon). PBMCs were activated with vehicle (PBS) or anti-CD3 (1 μg/mL) plus soluble anti-CD28 (1 μg/mL) mAbs (BD Pharmingen, San Jose, CA, USA). Then, cells pretreated with different doses of CL27c were incubated at 37°C and 5% CO_2 for 10 minutes. Akt phosphorylation was measured by western blot as described in the "Western blot analysis" in the Methods section.

Preparation and analysis of Treg Cells.

Cells were obtained from Lymph nodes and spleen from naive C57Bl/6J and purified with CD4 T cell isolation Kit (Miltenyi Biotec #130-104-454) and with CD25 biotin (eBioscience #13-0252-81) and microbeads anti-biotin (Miltenyi Biotec #130-090-485). Cells were then cultured for 72 hours in a 96-wells plate coated with anti-CD3 (1μg/ml, BD Bioscience #553057) and anti-CD28 (1 μg/ml, BD Bioscience #553294), in presence of TGF-β (1 ng/ml) or CL27c (1, 3, 10 or 30 uM). After 72 hours, cells were harvested and stained with anti-CD4 (BD Bioscience # 562891, used 1:200) and a fixable viability dye, APC-Cy7 (ebiosience # 65-0865-18, used 1:3000) diluted in PBS with 2% FBS for 10 minutes. Cells were subsequently fixed and permeabilized (eBioscience). After permeabilzation cells were stained with anti-FoxP3 (eBioscience, APC coniugated #17-5773-82, used 1:200) and processes for fax analysis (FACS VERSE). Positive stained cells were analyzed with FlowJo X software after removal of doublets events and debris through FSC-A/FSC-H gating.

Pharmacokinetic studies.

For pharmacokinetic analysis, all studies were conducted by the Istituto di Ricerche Biomediche (RBM) SpA – Merck Serono, Ivrea, Italy with the approval of their veterinarian service.

Statistical Analysis

Values are presented as mean ± standard error of the mean (SEM). One-way of two-way ANOVA followed by Bonferroni post-hoc test was used for parametric data, as appropriate, which were expressed as the mean ± standard error of the mean (S.E.M.). The non-parametric data were expressed as the median values and analyzed by Kruskal-Wallis followed by Dunn's test. The Mantel-Cox log rank test was used to determine differences between survival curves. Statistical significance was accepted when $P<0.05$. Statistical significance is indicated as: *$p<0.05$, **$p<0.01$, and ***$p<0.001$.
