# Supplementary material for: Inhalation of the prodrug PI3K inhibitor CL27c improves lung function in asthma and fibrosis
Source: Nat Commun. 2018 Dec 12;9:5232. doi: 10.1038/s41467-018-07698-6 (PMC6290777; doi:10.1038/s41467-018-07698-6)
Supplement: Supplementary file 3 — Reporting Summary [file 41467_2018_7698_MOESM3_ESM.pdf]

nature research

Corresponding author(s): Elisa Ciraolo

Reporting Summary

Nature Research wishes to improve the reproducibility of the work that we publish. This form provides structure for consistency and transparency in reporting. For further information on Nature Research policies, see Authors & Referees and the Editorial Policy Checklist.

Statistical parameters

When statistical analyses are reported, confirm that the following items are present in the relevant location (e.g. figure legend, table legend, main text, or Methods section).

n/a	Confirmed	
☐	☒	The exact sample size (n) for each experimental group/condition, given as a discrete number and unit of measurement
☐	☒	An indication of whether measurements were taken from distinct samples or whether the same sample was measured repeatedly
☐	☒	The statistical test(s) used AND whether they are one- or two-sided<br>*Only common tests should be described solely by name; describe more complex techniques in the Methods section.*
☒	☐	A description of all covariates tested
☐	☒	A description of any assumptions or corrections, such as tests of normality and adjustment for multiple comparisons
☐	☒	A full description of the statistics including central tendency (e.g. means) or other basic estimates (e.g. regression coefficient) AND variation (e.g. standard deviation) or associated estimates of uncertainty (e.g. confidence intervals)
☐	☒	For null hypothesis testing, the test statistic (e.g. F, t, r) with confidence intervals, effect sizes, degrees of freedom and P value noted<br>*Give P values as exact values whenever suitable.*
☒	☐	For Bayesian analysis, information on the choice of priors and Markov chain Monte Carlo settings
☒	☐	For hierarchical and complex designs, identification of the appropriate level for tests and full reporting of outcomes
☐	☒	Estimates of effect sizes (e.g. Cohen's d, Pearson's r), indicating how they were calculated
☐	☒	Clearly defined error bars<br>*State explicitly what error bars represent (e.g. SD, SE, CI)*

Our web collection on statistics for biologists may be useful.

Software and code

Policy information about availability of computer code

Data collection	Las X
Data analysis	Graphpad prism 7.0d, ImageJ 1.50i

For manuscripts utilizing custom algorithms or software that are central to the research but not yet described in published literature, software must be made available to editors/reviewers upon request. We strongly encourage code deposition in a community repository (e.g. GitHub). See the Nature Research guidelines for submitting code & software for further information.

Data

Policy information about availability of data

All manuscripts must include a data availability statement. This statement should provide the following information, where applicable:

- Accession codes, unique identifiers, or web links for publicly available datasets
- A list of figures that have associated raw data
- A description of any restrictions on data availability

Biochemichal data and imaging data are available upon request.

Field-specific reporting

Please select the best fit for your research. If you are not sure, read the appropriate sections before making your selection.

☒ Life sciences ☐ Behavioural & social sciences ☐ Ecological, evolutionary & environmental sciences

For a reference copy of the document with all sections, see nature.com/authors/policies/ReportingSummary-flat.pdf

Life sciences study design

All studies must disclose on these points even when the disclosure is negative.

Sample size	minimum of 3 biological replicates
Data exclusions	no data were excluded
Replication	all attempts to replicate were succesful.
Randomization	Random selection of sample from the population was performed
Blinding	Blinding was performed

Reporting for specific materials, systems and methods

Materials & experimental systems

n/a	Involved in the study
☐	☒ Unique biological materials
☐	☒ Antibodies
☐	☒ Eukaryotic cell lines
☒	☐ Palaeontology
☐	☒ Animals and other organisms
☒	☐ Human research participants

Methods

n/a	Involved in the study
☒	☐ ChIP-seq
☐	☒ Flow cytometry
☒	☐ MRI-based neuroimaging

Unique biological materials

Policy information about availability of materials

Obtaining unique materials	Unique Material used are available from author.

Antibodies

Antibodies used	Goat anti-Human IgM-MU chain specific antibody (Sigma-Aldrich #I0759-1MG) , rabbit anti-CD18 antibody (Acris Antibodies GmbH, Germany, Cat. Number B0842-1), biotinylated goat anti-rabbit antibody (Dako Agilent Pathology Solution, USA), phosphorylated Akt serine 473 (Cell Signaling Technology, Cat. Number 4060), pan Akt antibody (Cell Signaling Technology), GAPDH antibody (Cell Signaling Technology).
Validation	Were already validated in previous pubblications.

Eukaryotic cell lines

Policy information about cell lines

Cell line source(s)	CLL cells (# Hs 505.T, ATCC® CRL-7306™) , BMDMs were obtained from bone marrows isolated from femurs and tibias of C57Bl/6J mice, Neutrophils were obtained from bone marrow isolated from femurs and tibias of C57Bl/6J mice, PBMCs were isolated from blood, LL97a derived from a 48 years-old male (ATCC, Manassas, USA), CCD-16Lu obtained from a 35 years old male that died of astrocytoma –a disease unrelated to IPF - (ATCC), NHLF (#CC-2512; Lonza), DHLF-IPF (#CC-7231, Lonza, Switzerland).

Authentication	N/A
Mycoplasma contamination	Cells were negative for Mycoplasma and routinely tested.
Commonly misidentified lines (See ICLAC register)	N/A

Animals and other organisms

Policy information about studies involving animals; ARRIVE guidelines recommended for reporting animal research

Laboratory animals	C57BL/6J and Balb/c mice, males, 6-8 weeks.
Wild animals	N/A
Field-collected samples	N/A

Flow Cytometry

Plots

Confirm that:

☒ The axis labels state the marker and fluorochrome used (e.g. CD4-FITC).

☒ The axis scales are clearly visible. Include numbers along axes only for bottom left plot of group (a 'group' is an analysis of identical markers).

☒ All plots are contour plots with outliers or pseudocolor plots.

☒ A numerical value for number of cells or percentage (with statistics) is provided.

Methodology

Sample preparation	Cells were obtained from lymph nodes and spleen from naive C57BL/6. Then the cells were purified with CD4 T cell isolation Kit and with CD25 biotin and microbeads anti-biotin. After, the cells were cultured for 72 hours in a 96-wells plate with anti-CD3/CD28 coated (1 ug/ml), TGF-B (1 ng/ml) or Cl27C (1, 3, 10 or 30 uM). After 72 hours, the cells were harvested and stained with PBS (2% Bovine serum fetal) and anti-CD4 BV450 and fixable viability dye APC-Cy7 for 10 minutes. Then, the cells were fixed and permeabilized with intracellular fixation and permeabilization buffer set (eBioscience). At permeabilization process, anti-FoxP3 APC was used. After the staining, the cells were acquired with FACS VERSE machine and analyzed by FlowJo X software.
Instrument	FACS VERSE
Software	FACSDiva for data acquisition and FlowJo X for post-acquisition analyzes.
Cell population abundance	50.000 events
Gating strategy	Doublets were excluded by FSC-H and FSC-A gating (G1) and cell debris were excluded by SSC-A and FSC-A gating (G2) for all flow cytometry analysis. Proportion of viable cells was obtained by staining with fixable viability dye APC-Cy7.

☒ Tick this box to confirm that a figure exemplifying the gating strategy is provided in the Supplementary Information.
